# Impact of the secretome of activated pancreatic stellate cells on growth and differentiation of pancreatic tumour cells

**DOI:** 10.1038/s41598-019-41740-x

**Published:** 2019-03-28

**Authors:** Aseel J. Marzoq, Shakhawan A. Mustafa, Luzia Heidrich, Jörg D. Hoheisel, Mohamed Saiel Saeed Alhamdani

**Affiliations:** 1 Division of Functional Genome Analysis, Deutsches Krebsforschungszentrum (DKFZ), Im Neuenheimer Feld 580, D-69120 Heidelberg, Germany; 2 Kurdistan Institution for Strategic Studies and Scientific Research, Kurdistan Region, Sulaimaniya, Iraq

## Abstract

Pancreatic ductal adenocarcinoma (PDAC) exists in a complex desmoplastic microenvironment. As part of it, pancreatic stellate cells (PSCs) provide a fibrotic niche, stimulated by a dynamic communication between activated PSCs and tumour cells. Investigating how PSCs contribute to tumour development and for identifying proteins that the cells secrete during cancer progression, we studied by means of complex antibody microarrays the secretome of activated PSCs. A large number of secretome proteins were associated with cancer-related functions, such as cell apoptosis, cellular growth, proliferation and metastasis. Their effect on tumour cells could be confirmed by growing tumour cells in medium conditioned with activated PSC secretome. Analyses of the tumour cells’ proteome and mRNA revealed a strong inhibition of tumour cell apoptosis, but promotion of proliferation and migration. Many cellular proteins that exhibited variations were found to be under the regulatory control of eukaryotic translation initiation factor 4E (eIF4E), whose expression was triggered in tumour cells grown in the secretome of activated PSCs. Inhibition by an eIF4E siRNA blocked the effect, inhibiting tumour cell growth *in vitro*. Our findings show that activated PSCs acquire a pro-inflammatory phenotype and secret proteins that stimulate pancreatic cancer growth in an eIF4E-dependent manner, providing further insight into the role of stromal cells in pancreatic carcinogenesis and cancer progression.

## Introduction

Pancreatic ductal adenocarcinoma (PDAC) is the fourth most common cause of cancer death in Western Europe and North America and the number of deaths is bound to grow even further^1^. The dismal prognosis can be attributed to the disease’s ability to develop without early symptoms and its predisposition towards metastasis. All clinical trials to date have basically failed to introduce an overall more effective therapy. Surgery is still the most promising treatment option, but can only be applied to 10 to 20% of patients^2^. In addition, most patients experience recurrence. Difficulties in distinguishing PDAC from other diseases of the pancreas, such as chronic pancreatitis, make the exact diagnosis of PDAC even more complicated^3^. There is evidence that suggests a primary role and significant contribution of reactive stroma to pancreatic cancer lethality^4^. Indeed, stroma can form up to 90% of the tumour mass in PDAC^5^. The most common marker of reactive stroma in pancreatic cancer is the transformation of pancreatic stellate cells (PSCs) to myofibroblast-like cells. This cell type is characterised by the ability to express α-smooth muscle actin (α-SMA), an increased proliferation and secretion of fibrogenic cytokines^6^. In addition, PSCs are the major regulators of pancreatic fibrosis through their ability to release soluble factors and extracellular matrix protein (ECM) into the tumour microenvironment.

A significant body of evidence exists that reveals a bidirectional interaction between PSCs and PDAC. Cancer cells influence PSCs via mitogenic and fibrogenic factors which promote PSC activation, proliferation, migration and ECM remodelling capability^7,8^. In return, PSC activation leads to the production and secretion of different paracrine stimulants and growth factors, such as platelet-derived growth factor (PDGF), transforming growth factor beta (TGF-β1) as well as cytokines, such as the inflammatory mediators interleukin-1-beta (IL-1ß), interleukin-6 (IL-6), interleukin-8 (IL-8), and tumour necrosis factor alpha (TNF-α)^9^. In addition, several investigators have reported that PSCs support tumour development as well as chemoresistance^10,11^. PSCs not only increase tumour cell viability *in vitro*, but also inhibit apoptosis, increase tumour invasion and promote metastasis *in vivo*^10–12^. In fact, a complex interaction between PSCs and pancreatic cancer cells has been suggested, although the molecular basis of this interaction is yet to be discovered. Several studies have shown that secreted proteins from activated PSCs are associated with cancer progression^13–15^.

Recently, several proteomics technologies have been utilised to investigate cancer cell secretomes, like those of breast^16^, lung^17^ and pancreas^18^, for example. However, the significance of interaction between tumour and surrounding cells has rarely been addressed at the proteome level. Pancreatic cancer is an ideal example for studying the impact of the microenvironment on tumour development and progression. Much of the molecular interaction and communication between PDAC cells and their cellular microenvironment is probably still unknown. Accordingly, a more global view at the proteome shared between PDAC and its cellular microenvironment, as in part represented by PSCs, may help in unravelling the identity of molecules used for communication. PDAC and PSCs represent two cell types that are central in such interaction. Analysing their molecular communication could contribute to monitoring or even controlling cancer eventually.

**Figure 1:**
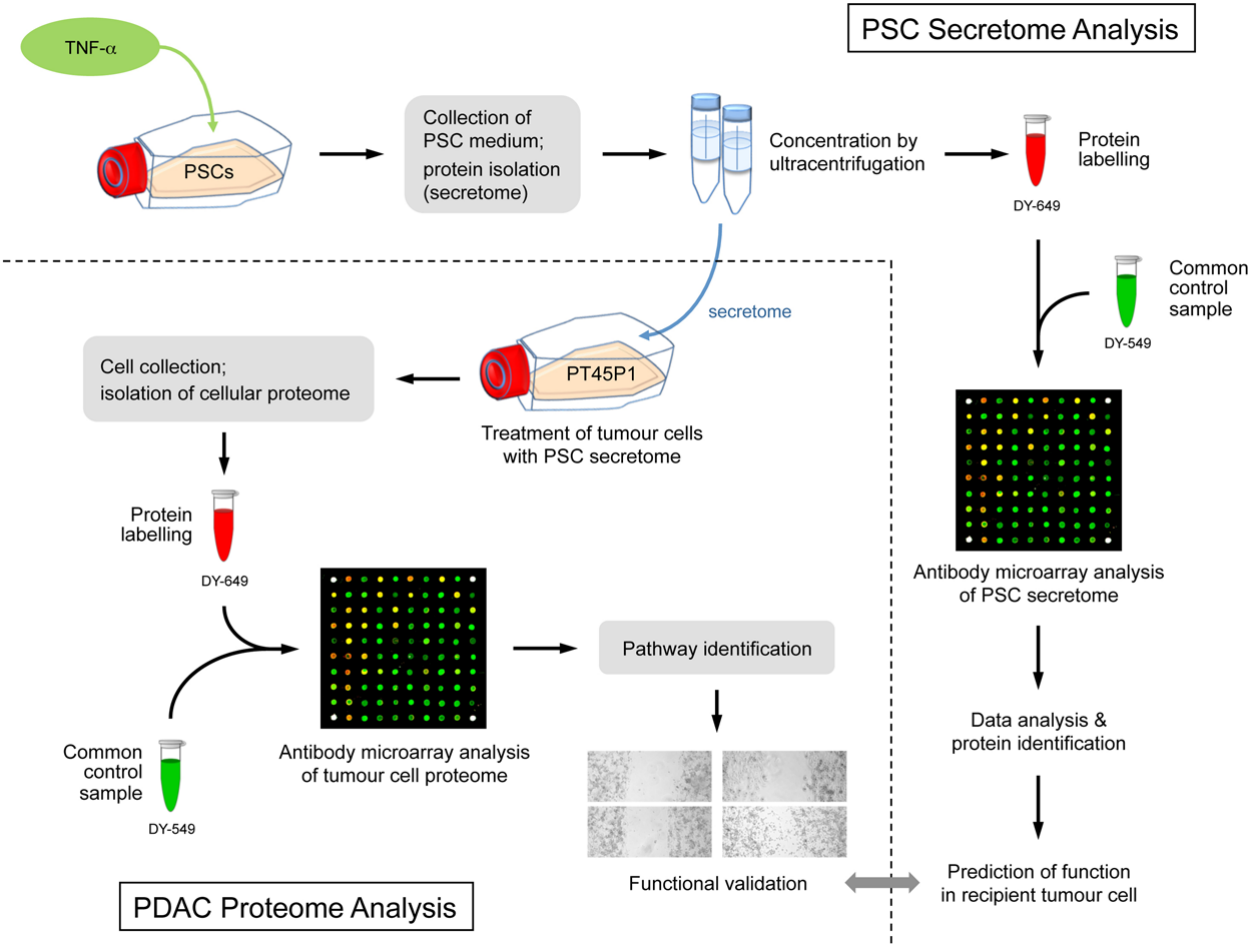
Scheme of the overall experimental set-up. First, the protein content of the secretome of activated PSCs was analysed. On this basis, predictions were made about the functional consequences, which the secreted proteins would have in recipient cells. Second, PT45P1 tumour cells were grown in media conditioned with secretome. The intracellular proteome was studied and again used for functional predictions. Both the predictions from secretome and intracellular proteome were compared and validated by investigating the actual functional variations observed and by identifying relevant regulative factors.

This study aimed at profiling the secretome isolated from activated PSCs in order to look for proteins that may affect tumour cells and are specific to the secretome of activated PSCs. The identified protein patterns were used for a prediction of functional consequences they may have in recipient tumour cells. Subsequently, tumour cells were grown in media conditioned with PSC secretomes. The proteome and mRNA profiles of the PDAC cells were studied and compared to the profiles obtained from cells grown without any secretome supplement in the medium. The identified proteins and relevant processes affect cell apoptosis, proliferation and metastasis. With the eukaryotic translation initiation factor 4E (eIF4E), a protein was found whose expression was triggered by conditioning with secretome of activated PSCs and which is involved in the control of many of the identified protein variations in the tumour cells. This regulatory process was confirmed by functional studies. The overall experimental and analytical workflow is presented in Fig. 1.

## Experimental Procedures

### Materials

All materials used in this study were purchased from Sigma-Aldrich (Taufkirchen, Germany) unless stated otherwise and were of highest purity or protein grade. In the analysis, a set of 810 antibodies was used as reported earlier^19^. Their target proteins and origin are listed in Suppl. Table. S1.

### Experimental design and statistical rationale

The protein content of the secretome of activated and non-activated PSCs was studied by incubations on complex antibody microarrays. From the observed binding patterns, significant differences were defined and applied to predict functional changes that the secretome from activated PSCs could trigger in recipient tumour cells. The prediction was followed up by experiments, analysing in detail the changes occurring at the protein level within tumour cells grown in presence of secretome, again by means of antibody microarrays. These intracellular protein level variations were utilised to identify affected functional pathways. The relevance of this was confirmed by studying tumour cell functions at appropriate experimental conditions. In this process, a translational initiation factor was found to act as a central regulatory element. Its relevance to the observed changes was confirmed by targeted functional studies.

### Culture of PSCs and collection of conditioned media

Human immortalized PSCs were a kind gift from Ralf Jesenofsky (Mannheim University Hospital, Germany). The PDAC cell line PT45P1 was kindly provided by Aldo Scarpa (Verona, Italy). Cells were grown in 175 cm^2^ flasks in Iscove’s Modified Dulbecco’s Medium (IMDM) (Invitrogen, Carlsbad, USA) supplemented with 10% heat-inactivated foetal bovine serum (CSL, Melbourne, Australia) in the presence of 50 U/ml penicillin and 50 µg/ml streptomycin in a 5% CO_2_ incubator at 37 °C. At 90% confluence, PSCs were washed twice with serum-free medium and once with phosphate buffered saline (PBS). The cells were incubated with serum-free IMDM medium for 24 h to synchronize cell growth. The medium was then removed and replaced by serum- and phenol-red-free IMDM with or without 10 ng/ml TNF-α. Each condition was done in quintuplicate. After 48 h incubation, about 20 ml of conditioned medium was collected from each flask. It was centrifuged at 3000 g for 10 min to remove cell debris. The supernatant was concentrated to 200 μl using a 3 kD Amicon ultra centrifugal filter device (Millipore Billerica, Darmstadt, Germany), followed by the addition of 20 ml 0.1 M bicine, pH 8.5, as exchange buffer and another concentration to 200 μl. Protein concentration was determined with the Bicinchoninic Acid Protein Assay Reagent kit (Thermo Scientific, Bonn, Germany). The concentration was adjusted to 1.0 mg/ml using the bicine buffer as diluent.

### Cancer cell growth in conditioned medium

PT45P1 cells were incubated with serum-free IMDM medium (SFM) for 24 h. SFM was removed and PT45P1 cells were replenished with new serum- and phenol-red-free IMDM alone (control) or medium supplemented with either 100 µl concentrated supernatant from PSCs treated with TNF-α (activated PSCs) or untreated PSCs (non-activated PSC). After growth for 48 h, the PT45P1 cells were washed three times with ice-cold PBS and subjected to protein extraction as described below. All experiments were done in quintuplicates. Additionally, to insure that incubation for 48 h in serum-free medium was not affecting cell viability, cell growth was monitored at 24, 48 and 72 h for PSCs and PT45P1 cells grown in serum-free media (negative control). Viability of cells was 95% or higher at all tested time points.

### Protein extraction

Protein extraction was performed as described previously^20^. In brief, after washing with ice-cooled Dulbecco’s modified phosphate buffered saline (DPBS, Thermo Scientific), the PT45P1 cells were layered with a minimal volume of extraction/labelling solution composed of 50 mM bicine buffer, pH 8.5, 20% glycerol, 1.0 mM MgCl_2_, 5.0 mM EDTA, 1.0 mM phenylmethanesulfonyl fluoride, 1.0 IU/ml benzonase (Merck Bioscinces, Schwalbach, Germany), Halt Protease and Phosphatase Inhibitor Cocktail (Thermo Scientific), 0.5% Nonidet P-40 substitute, 1.0% cholic acid, 0.25% n-dodecyl-β-maltoside (GenaXXon Bioscience, Ulm, Germany), and 0.5% amidosulfobetaine-14. Flasks were kept on ice for 30 min with occasional shaking. Cell lysates were collected with a cell scrapper followed by centrifugation at 20,000 g and 4 °C for 20 min. The supernatant was collected and the protein concentration was determined with the Bicinchoninic Acid Protein Assay Reagent kit (Thermo Scientific).

### Protein labelling

Protein labelling was performed as described^21,22^. In brief, the protein concentration was adjusted to 1.0 mg/ml for the cell secretomes and to 2.0 mg/ml for cell lysates. The fluorescent NHS-ester dyes DY-649 or DY-549 (Dyomics, Jena, Germany) were then added to each sample at a molar ratio dye/protein of 7.5.

### Antibody microarray

The microarrays were produced as described in much detail earlier^21,22^. Briefly, the antibodies were spotted from 384-well plates onto epoxysilane-coated slides (Nexterion-E; Schott, Jena, Germany) using the contact printer MicroGrid-2 (BioRobotics, Cambridge, UK) and SMP3B pins (Telechem, Sunnyvale, USA) at a humidity of 50–60%. The spotting buffer was prepared by generating 15 µl in each well of 50 mM bicine buffer, pH 8.5, containing 1.0 mM MgCl_2_, 5% trehalose, 0.005% Tween-20 and 5 µg of the respective antibody. On each slide, the antibodies were printed in quadruplicates. After printing, the slides were equilibrated at a humidity of 50–60% overnight and stored in dry and dark conditions at 4 °C until use.

### Protein profiling

The protein samples were investigated on antibody microarrays as described in detail before^23^. All steps were performed in the dark. Before incubation with labelled samples, slides were washed twice (for 10 and 5 min, respectively) with PBS containing 0.05% Tween-20 (PBST). Following washing, the slides were blocked with 10% non-fat dry milk (Biorad, Munich, Germany) in PBST at room temperature for 3 h. Each slide was incubated with 35 μg of DY-649 labelled sample and 35 μg of a common reference sample made of the pooled protein extracts of 23 PDAC cell lines labelled with DY-549 (Suppl. Table. S2)^19^. Incubation was done using Quadriperm chambers (Greiner Bio-one, Frickenhausen, Germany) in 5 ml of PBST supplemented with 10% milk at 4 °C overnight. The slides were washed 4 times for 5 min each with PBST, rinsed with deionized water and dried in a ventilated oven at room temperature. Scanning of the slides was performed using a Tecan power scanner (Tecan, Grödig, Austria) at constant laser power and PMT. Image acquisition and analysis were performed with GenePix Pro 6.0 software (Molecular Devices, Sunnyvale, USA), generating numerical values of signal intensities.

### Data analysis

The signal intensities from microarray experiments were analysed with the Chipster software^24^. The median of signal intensity with local background for each spot at both red (DY-649) and green (DY-549) channels was used to generate ratios. Ratios were normalized using the Loess method with background correction offset [0, 50] of the *normexp* method^25^. Test of significance between control and treatment groups was performed using the Empirical Bayes test with Bonferroni-Hochberg adjustment of p-values^26^. The empirical Bayes make use of a moderated t-statistic in which posterior residual standard deviations are applied rather than ordinary standard deviations, which give a far more stable inference when the number of arrays is small^26^. A p-value of 0.05 or less was considered significant. Multiple-set Venn diagrams were generated using the open-source software VENNTURE^27^. The bio-functional annotation of the differentially expressed proteins was performed with the Ingenuity Pathways Analysis (IPA) software (version 6.3; Ingenuity Systems, Redwood City, USA). Prediction of variations in biological functions was performed using a z-score of +2 or −2, respectively, as threshold for significance. Protein functional interaction networks were evaluated using the open-source software STRING 9.0^28^. For the proliferation assay, unpaired student t-test (two-tailed) was used to determine the significance of differences between the control (serum-free incubations) and each of the other treatments. The inter- and intra-assay coefficient of variance (CV) was always less than 20%.

### Cell transfection

We used the siRNA gene silencer system (siRNA #6554) as well as a control siRNA (#6568) of Cell Signaling Technology (Danvers, USA) to perform the *EIF4E* gene silencing in the pancreatic cancer cell lines PT45P1, Panc-1 and Capan-1 according to the manufacturer’s protocol. Briefly, RNA transfections were carried out in 6-well or 96-well plates using siPORT NeoFXTM (Ambion, Carlsbad, USA) reagent. siPORTTM NeoFXTM transfection agent and the RNA molecules were mixed and distributed on the culture plates and overlaid with the cells. The final transfection volume in a 6-well plate was 2.5 ml of medium containing 2 × 10^5^ cells per well; in 96-well plates, it was 100 μl of medium containing 5 × 10^3^ cells per well. The final concentration of the RNA molecules transfected was 100 nM. After this procedure, the plates were maintained at 37 °C and 5% CO_2_. After 48 h, cells were serum-starved overnight and either left untreated or treated with activated PSC secretome for 24 h.

### ELISA

To determine the concentration of fibronectin and collagen, 100 μl PSC culture supernatant (20 µg/ml) were coated onto 96-well microtiter plates (Nunc-Maxi Sorp, Langenselbold, Germany) in five replicate experiments and incubated overnight at 4 °C. Subsequently, the plates were blocked with 5% non-fat milk in PBST for 3 h prior to an incubation overnight at 4 °C with polyclonal rabbit-anti-human-collagen type I (Biomol, Hamburg, Germany) or polyclonal rabbit-anti-human fibronectin antibody. Wells were washed with PBST and incubated with HRP-conjugated secondary antibody (Santa Cruz Biotechnology, Germany). Antibody complexes were detected with the peroxidase substrate SureBlue TMB (KPL, Gaithersburg, Germany). Plates were read on a standard plate reader at 540 nm.

### Western blotting

Confirmations of PSC secretome proteins and PT45P1 cell lysate proteins were obtained by Western blot analyses. Briefly, PSCs, PT45P1 and Panc-1 cells were cultured, treated and collected as described above. Equal amounts of protein from each secretome or lysate sample were diluted in a reducing sodium-dodecyl-sulfate polyacrylamide gel sample buffer, heated to 96 °C for 5 min and separated by electrophoresis on a 6, 10 or 12% SDS-polyacrylamide gel (SDS-PAGE). Resolved proteins were transferred to nitrocellulose membranes (VWR International, Darmstadt, Germany). Efficient protein transfer to the membrane was routinely confirmed by the reversible staining of membranes with Ponceau S dye solution (SERVA Electrophoresis, Heidelberg, Germany). Membranes were washed and blocked for 1 h at room temperature with 5% non-fat dry milk in PBST. After blocking, the membrane was incubated with the 1:500 diluted primary antibody at 4 °C overnight. After incubation with a 1:10000 dilution of peroxidase-conjugated anti-rabbit secondary antibody (Santa Cruz Biotechnology), proteins were visualised by using the ECL kit (Amersham Biosciences, Freiburg, Germany). Negative control plots were probed using non-immune IgG (Cell Signaling Technology). Levels of other proteins were studied accordingly using the following primary antibodies: antibodies targeting collagen (Coll), IL-1ß, fibroblast growth factor 1 (FGF-1), interleukin-4 (IL-4), plasminogen activator inhibitor 1 RNA-binding protein (SERPINE), BAX, CDKN2A, CAPS-9 and NFKB-1 were from Santa Cruz Biotechnology (Texas, USA); Enolase (ENO1): abcam (Cambridge, UK); IMPDH and eIF4E: Cell Signaling Technologies; TNF-α: Peprotech (Hamburg, Germany); fibronectin (FN1), c-JUN and CCNA2: Acris (Herford, Germany); GAPDH and α-tubulin: Sigma-Aldrich.

### Proliferation assay

Changes in proliferation rates of cancer cells treated with PSC secretome were determined using the CyQUANT NF Cell Proliferation Assay kit (Invitrogen, Schwerte, Germany). The assay is based on the measurement of cellular DNA content via binding of a fluorescent dye. PT45P1 were seeded in 96-well flat bottom tissue culture plates at a density of approximately 5000 cells/well and allowed to attach for 5 h. Then cells were washed twice with PBS and incubated in SFM for 24 h. SFM was removed and cells were incubated for 48 h with SFM alone or medium conditioned with concentrated secretome of TNF-treated PSCs. After 48 h incubation, the medium was removed and cell proliferation was determined according to the manufacturer’s recommendations. Briefly, 100 µl of the CyQUANT NF reagent was added to each well, and the plate was incubated at 37 °C for 1 h. Absorbance measurements were done at an excitation of 485 nm and emission at 530 nm. For transfected cells, cell proliferation was quantitated by using the cellTiter-Glo Luminescent cell viability assay kit (Promega, Madison, USA). Briefly, 5 × 10^3^ PT45P1 and Panc1 cells were plated in 96-well plates and allowed to adhere. After transfected with siRNA and treated as above for another 24 h, cell proliferation was determined according to the instructions of the manufacturer.

### Cell migration assay

Cell migration was assayed using the *in vitro* wound healing method. For this assay, PT45P1 cells were placed in 6-well plates and transfected with or without 100 nM of *EIF4E* siRNAs for 48 h as described above. Cells were serum-starved overnight. A wound was created on the cells by scraping a gap using a sterile micropipette tip followed by washing the cell monolayer with PBS three times. The cells were then treated with PSC secretome as described in the cell proliferation assay section above. Mitomycin C (0.02 mg/ml) was added to inhibit proliferation. ImageJ (http://dev.mri.cnrs.fr/projects/imagej-macros/wiki/Wound_Healing_Tool) was used to quantify the migration assay from five different locations of three replicate experiments.

### Apoptosis assay

The effect of various conditions on PT45P1 cell apoptosis was conducted by measurement of a mitochondrial membrane potential assay using the Mito-ID Membrane Potential kit of Enzo Life Sciences (Lörrach, Germany). Briefly, PT45P1 cells were seeded at 2 × 10^4^ cells per well and allowed to attach overnight. After that, cells were treated either with conditioned medium supplemented with supernatant from activated PSCs or inactivated PSCs, respectively. After 48 h, the Mito-ID Membrane Potential assay was used according to the manufacturer’s instructions. A decrease in mitochondrial membrane potential is considered to be a function of accelerated apoptotic activity. For transfected cells, cell apoptosis was assessed using the Caspase-Glo3/7 assay kit (Promega) according to the manufacture’s instruction. Briefly, after Panc-1 and PT45P1 cells had been transfected and treated as above, the dye solution was added to cells and incubation continued at 37 °C for 30 min. Cell apoptosis was determined by measuring the differences in luminescence.

### Real-time qPCR

Analysis of mRNA expressions was accomplished by RT-qPCR using primers from Qiagen (Hilden, Germany). Total RNA was extracted from PT45P1 cells using the Trizol Reagent (Invitrogen) following the manufacturer’s instructions. The cDNA was synthesised using the protoscript first strand cDNA synthesis kit (Cell Signaling Technologies). A total of 2 mg of total RNA was reverse transcribed using a SYBR Green PCR Kit (Invitrogen). For all steps, the samples were kept on ice. Following the guidelines of the manufacturer, PCR was performed at 95 °C for 10 min, followed by 50 cycles of 15 s at 95 °C and 1 min at 60 °C. The reactions were performed in triplicate.

## Results

### The effect of TNF-α on the PSC secretome

**Figure 2:**
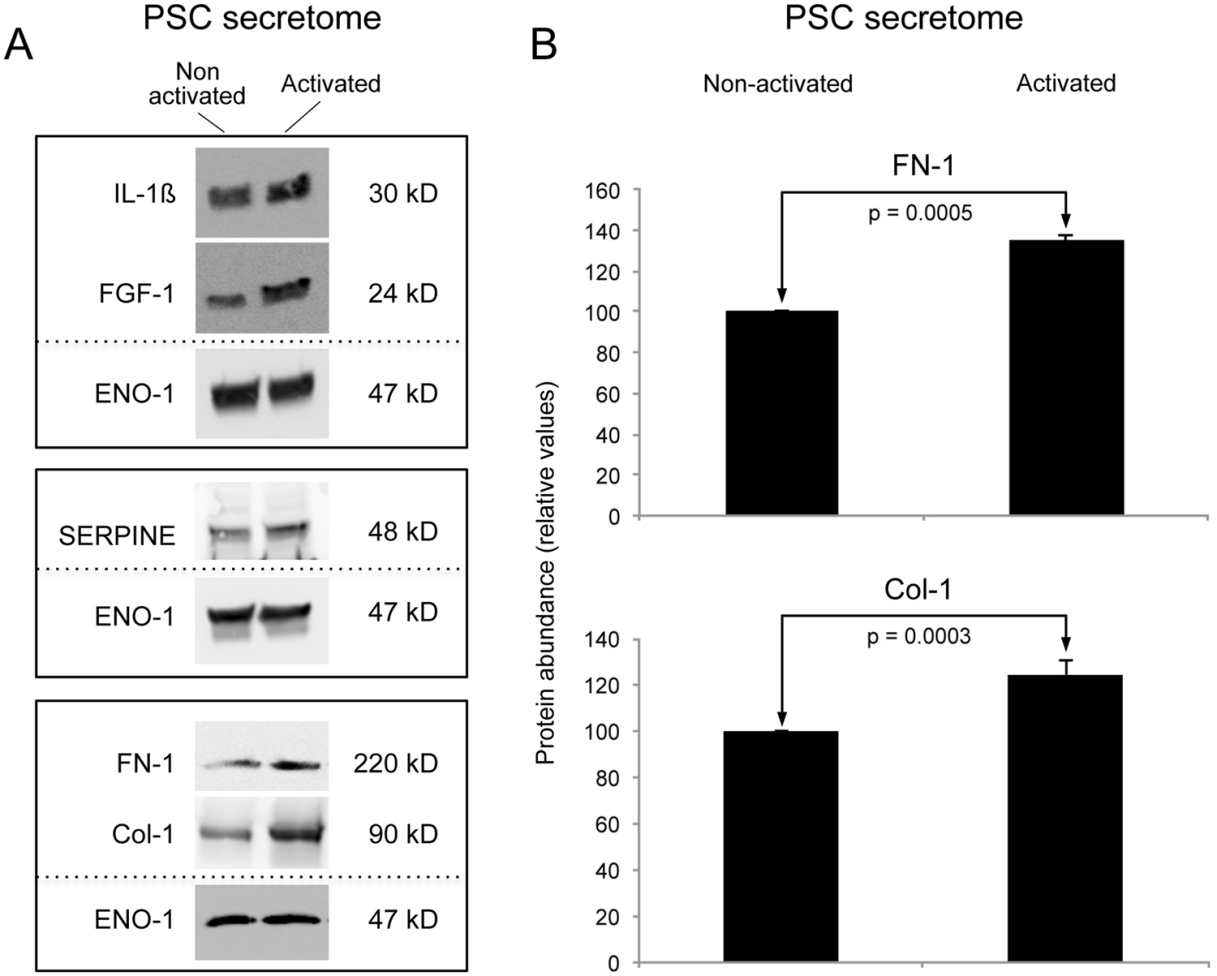
Verification of PSC secreted proteins by immunoblot and ELISA. (**A**) Western blotting of conditioned media concentrates from activated or quiescent PSCs; enolase (ENO-1) was used as control protein. (**B**) Bar chart of the relative abundance in secretome of non-activated or activated PSCs, respectively, of fibronectin 1 (FN-1) and collagen-1 (Col-1); detection was by commercial ELISA.

We had previously examined the effect of pro-inflammatory factors on the biological functions of PSCs, taking advantage of the very antibody microarray utilised here. TNF-α was identified as the prime factor responsible for PSC activation^6^. Consequently, we used TNF-α in this study accordingly. The secretome released by TNF-α activated PSCs was collected and analysed on antibody microarrays. To this end, the growth media of activated and non-activated PSCs were collected individually in three independent technical replicates each. Only serum-free media were used for the experimental set-up in order to avoid any interference from growth factors and cytokines contained in added serum. Even low concentrations would be detected by the high sensitivity of the affinity-based antibody microarray assay. In addition, signal variations could have arisen from the effect of serum-soluble factors on the PSCs. In the secretome of TNF-α treated PSCs, 309 secreted proteins were found to be differentially abundant in the cell culture medium compared to samples from non-activated cells (Suppl. Table. S3). Only 18% of the identified proteins were known to be secreted via classical secretory mechanism, and 17% were categorized as plasma membrane proteins (Suppl. Fig. 1). Particular protein abundance variations detected by the antibody microarray analyses were validated by Western blot or ELISA, or both. In accordance with the microarrays data, the levels of proteins SERPINE, IL-4, IL-1ß, and FGF-1, for example, were found to be increased in the secretome of activated PSCs. The same was observed for ECM proteins, such as FN-1 and Col-1 (Fig. 2), which are known to be preferentially expressed by activated PSCs and reported to be involved functionally in cell proliferation and angiogenesis^29^. A bio-functional annotation of the 309 proteins (Suppl. Table. S4) indicated the involvement of a large number of proteins in cancer and tumour-associated fibroblast processes as well as a close association with cancer-related functions, such as cell apoptosis, cellular growth and proliferation (Table 1). In addition, cell mobility aspects were strongly represented, such as metastasis, cellular movement and adhesion of eukaryotic cells. Many ECM proteins were regulated in the secretome of TNF-α activated PSCs, including matrix-metalloproteases (MMPs) and tissue inhibitor of metalloproteases (TIMPs). Examples are FN-1, MMP-10, MMP-11 and MMP-14, whose concentrations were significantly elevated. To predict potential activities of the secreted proteins upon uptake by recipient tumour cells, a network map was constructed on the basis of the secretome protein profile using the IPA software (Suppl. Fig. 2). The predicted cellular functions triggered by the protein variations observed in the PSC secretome include activation and proliferation of fibroblasts, proliferation and migration of tumour cells as well as invasion. In addition, apoptosis of cancer cells was predicted to be strongly inhibited.

**Table 1:** Functional analysis of the antibody microarray data obtained from PSC secretome upon TNF-α treatment.

Functional Annotation	p-Value	Predicted Activation State	z-Score	Molecule Number	Typical Molecules
Invasion of cells	1.21E-23	Increased	2.952	33	CCL11, CSF2, CTGF, FGF2, FN1, IFNG, IGF1, IGF2, IL1B, IL6, TIMP1, TIMP2, TNF, VEGFA, VEGFB, VEGFC, VTN
Stimulation of connective tissue cells	3.14E-19	Increased	2.951	12	CSF2, FGF2, IFNG, IGF1, IL10, IL1B, IL4, IL6, TNF, VEGFA
Generation of reactive oxygen species	1.22E-13	Increased	2.884	15	ALB, CSF2, IFNG, IGF1, IL10, IL1B, IL6, MMP14, SERPINB5, TNF
Migration of cells	1.54E-39	Increased	2.876	58	CCL11, CCL4, CSF2, CTGF, FGF1, FGF2, FN1, IGF1, IL4, IL6, MMP10, MMP11, MMP12, MMP14, TGFBI, THBS2, TIMP1, TIMP2, TNF, VEGFA, VEGFB, VEGFC, VTN
Proliferation of fibroblasts	8.27E-14	Increased	2.747	17	CTGF, DCN, FGF1, FGF2, FN1, IGF1, IGF2, IGFBP3, IL1B, IL4, IL6, TNF,
Growth of tumour	3.50E-31	Increased	2.745	38	CCL11, CSF2, CTGF, FGF1, FGF2, FN1
Proliferation of lymphoma cell lines	1.08E-13	Increased	2.739	13	CCL11, IFNG, IGF1, IGF2, IGFBP1, IGFBP3, IL10, IL15, IL2, IL4, IL6, TF, TNF
Activation of fibroblasts	6.06E-14	Increased	2.563	8	ENO1, IFNG, IGF1, IGFBP3, IL6, VEGFB
Invasion of tumour cell lines	3.90E-18	Increased	2.515	25	CCL11, FGF2, FN1, MMP14, SERPINB5, TIMP1, TIMP2, TNF, VEGFA, VEGFB
Growth of malignant tumour	6.38E-24	Increased	2.412	27	FGF1, FGF2, GRN, IL10, IL12A, IL15, IL1AIL6, KLK3, TNF,
Cell movement of tumour cell lines	1.63E-26	Increased	2.370	35	AREG, CCL11, CCL4, CSF2, CTGF, CXCL10, CXCL8, CXCL9, DCN, FGF1, FGF2,
Migration of tumour cell lines	4.10E-27	Increased	2.364	33	AREG, CCL11, CSF2, CTGF, CXCL10, CXCL8, MMP14, SERPINE1, TGFBI, THBS2, TIMP1, TIMP2, TNF, VEGFA
Proliferation of tumour cell lines	1.58E-24	Increased	2.339	42	IL10, IL12A, IL1B, IL1RN, IL2, IL32, INS
Mitogenesis	1.18E-22	Increased	2.315	19	FGF1, FGF2, GRP, IGF1, IGF2, IGFBP2
Proliferation of antigen presenting cells	1.11E-17	Increased	2.225	13	ENO1, EWSR1, FGF1, FGF2, FLNA, FN1, CSF2, DCN, IFNA1/IFNA13, IFNG, IGF1, IL10
Formation of cells	8.35E-15	Increased	2.219	27	ADCYAP1, ALB, AREG, BGN, CCL11, CCL4, CSF2, CTGF, CXCL10
Proliferation of cells	3.78E-24	Increased	2.150	59	FGF2, FN1, FRZB, GRN, GRP, GSN, IFNA1, IFNA13, IFNG, IGF1, IGF2, IGFBP1, IGFBP2, IGFBP3, IL10, IL12A, IL15, IL1A, IL1B, IL1RN, IL2, IL32, IL4
Activation of cells	8.99E-30	Increased	2.142	41	TF, TG, TIMP1, TNF, TNFSF14, VEGFA, VTN
Proliferation of tumour cells	2.73E-34	Increased	2.130	35	IGFBP3, IL10, IL12A, IL15, IL1A, IL1B, IL1RN, IL2, IL32, IL4, IL6, KLK3
Proliferation of immune cells	2.18E-20	Increased	2.042	30	ALB, CCL11, CCL4, CSF2, CTGF, CXCL10
Proliferation of leukocyte cell lines	8.60E-20	Increased	2.032	18	CSF2, FGF1, FGF2, FN1, IFNG, IGF1
Proliferation of cancer cells	5.56E-22	Increased	2.020	24	AREG, CSF2, CXCL8, DCN, FGF1, FGF2, GRN
Apoptosis of cancer cells	−1.27E-14	Decreased	−2.578	15	ALB, CSF2, FGF2, IFNG, IGF1, IGFBP3, IL10, IL15, IL4, IL6, MMP11, TF, TNF, VEGFA, VEGFC
Apoptosis of tumour cells	−8.67E-21	Decreased	−2.882	21	FGF2, IFNG, IGF1, IGFBP3, IL10, IL15, IL2, IL4, IL6, KLK3, MMP11, TF, TNF, VEGFA, VEGFC
Necrosis of tumour	−1.42E-24	Decreased	−3.598	26	IL1B, IL2, IL4, IL6, KLK3, MMP11, SERPINB5, SERPINE1, TF, THBS2, TIMP1, TIMP2, TNF, VEGFA, VEGFC
Cell death of tumour cells	−1.92E-23	Decreased	−3.598	25	CSF2, CXCL8, FGF2, IFNG, IGF1, IGFBP3, IL10, IL15, IL1A, IL1B, IL2, IL4, IL6, KLK3, MMP11, SERPINB5, SERPINE1, TF, THBS2, TIMP1, TIMP2, TNF, VEGFA, VEGFC

### The effect of PSC secretomes on tumour cells

For elucidating the actual effect of PSC secretome on tumour cells, pancreatic adenocarcinoma PT45P1 cells were grown in presence of secretome from either activated PSCs (PT45P1-act) or non-activated PSCs (PT45P1-non-act). In addition, cells were grown in absence of any substitution (PT45P1-ctrl). Cell viability was tested throughout and was at 95% to 100% for all cultures at any time. Prior to using supernatants of PSC cultures for conditioning media of tumour cell cultures, the supernatants were subjected to a buffer exchange step during the ultrafiltration process that was applied for increasing protein concentration. Only by this measure we could prevent the accumulation of small molecules, such as metabolites or other cellular by-products, which might have influenced cell function in the tumour cell cultures. After growth for 48 h, the PT45P1 cells were collected and washed to remove any secretome contamination. Subsequently, their cellular proteomes were isolated. Compared to PT45P1-ctrl, a substantial number of proteins differed significantly in abundance in all secretome-treated PT45P1 samples (Suppl. Tables S5, S6). This indicates that PSCs – not surprisingly – affect neighbouring tumour cells by their secretome. For the identification of differences that are due to the activation state of the PSCs, from which the secretomes were collected, the PT45P1-act and PT45P1-non-act protein samples were compared, yielding 247 cellular proteins that differed in abundance between them (Suppl. Table. S7). They mainly cluster in 12 functional categories, which are associated with processes such as metabolism of reactive oxygen species, cell death, cancer cell apoptosis and differentiation (Table 2).

**Table 2:** Prediction of cellular functions that triggered in PT45P1 tumour cells by the secretome of activated PSCs compared to non-activated PSCs. Proteins that varied are listed.

Biological Functions	p-Value	Molecules	Molecule Number
Carcinoma	1.44E-25	ADAM9, AKT3, ALB, APC, APEX1, AREG/AREGB, AURKB, BAX, BRCA1, BUB1, CCL5, CCNA2, CCNB1, CDH13, CDK4, CDKN1A, CDKN1C, CDKN2A, CDKN2B, CKB, CLDN16, COL1A1, CTSD, CXCL10, CYP2C8, DKK1, EDNRA, EDNRB, EEF1A1, ENO2, EP300, ETS2, EWSR1, FAS, FGF2, FN1, FOLR1, FUBP1, GAPDH, GAS1, GJB1, GNAS, GRP, GSTP1, HMMR, HSPA8, IFI27, IFITM2, IFNA1/IFNA13, IFNG, IFNGR1, IGF1, IGF2, IL10, IL15, IL1B, IL4, IL6, IL8, JUN, KDM5A, KDR, KLF8, LGALS4, LIFR, LMNA, MAGED2, MAPK10, MCM5, MGMT, MIF, MLH1, MMP11, MMP13, MOXD1, MUC2, MXI1, MYBL2, NCL, NOS2, OVGP1, PCNA, PGF, PRDX2, PRKCG, PSMF1, PTK2, RARB, RASSF1, RB1, RPL7, RPS19, RUNX3, S100A4, S100A6, S100A8, S100A9, SDC1, SELE, SERPINE1, SERPINE2, SFRP2, SLC29A1, SLC7A5, SOCS1, SOD1, SPP1, TF, TFPI2, TGFBI, TGFBR2, TIMP1, TIMP2, TNF, TNFSF13, TRAPPC11, TSPAN8, TUBA1A, TUBB, VDR, VEGFA, VEGFC, ZC3H13	123
Binding of DNA	1.35E-23	AIFM1, ALB, BAX, CCL11, CCL5, CDKN1A, FAS, FCER1A, FGF2, FN1, FPR1, GNAS, IFNG, IGF1, IL10, IL13, IL1B, IL2, IL32, IL4, IL6, IL8, NOS2, NR4A1, PRDX2, RB1, S100A6, SELE, SOD1, TF, TNF, VDR	51
Apoptosis of pancreatic cancer cell lines	3.79E-13	BAX, CASP9, FAS, FGF2, IFNG, JUN, NR4A1, TNF	14
Induction of lipid	1.88E-11	ALB, APEX1, BRCA1, CALR, CDKN1A, CDKN2A, EP300, ETS2, FAS, FGF2, FN1, GNAS, GRP, GSTP1, IFNA1/IFNA13, IFNG, IGF1, IL10, IL13, IL15, IL1A, IL1B, IL2, IL4, IL6, IL8, IRF7, JUN, KDR, LMNA, MAPK3, MGMT, MIF, NFKB1, NFKB2, NR4A1, PCNA, POU2F1, POU5F1, RARB, RASSF1, RB1, SOCS1, SOD1, TF, TNF, TNFAIP3, VDR, VEGFA, YBX1, ZBTB17	9
Proliferation of cervical cancer cell lines	2.70E-11	C1QC, CDKN1A, CSF2RB, IFNA1/IFNA13, IFNG, IL10, IL13, IL15, IL1A, IL4, IL6, IRF7, SOCS1, TNF	19
Metabolism of reactive oxygen species	1.02E-10	CCL5, FN1, IFNG, IL10, IL2, IL4, IL6, SELE, TIMP2, TNF, VEGFA	32
Differentiation of cells	7.70E-09	FGF2, IL1A, IL1B, TIMP1, TNF	14
Synthesis of nitrite	1.31E-08	FGF2, GRP, IL10, IL15, IL1A, IL1B, IL6, MIF, TNF	8
Apoptosis of myofibroblasts	1.77E-08	BAX, CDKN2A, FAS, BCL2L2, IFNG, IGF1, IL1B, IL32, IL6, IL8, RPS19, S100A4, SOCS1, TNF	5
Migration of monocytes	2.04E-08	ACTB, APEX1, ARID4A, AURKB, CDKN1A, CDKN2A, DKK3, EP300, EWSR1, FOLR1, IFNG, IGF1, IGF2, NCL, RB1, SOCS1, SPP1, TNF, TUBB	11
Apoptosis of carcinoma cells	3.84E-08	ALB, FGF2, IFNG, IGF1, IL4, IL6, IL10, TNF	8
Cell death of pancreatic cancer cell lines	3.84E-08	IFNG, IL13, IL1A, IL1B, IL6, MTA1, NOS2, TNF	8

**Figure 3:**
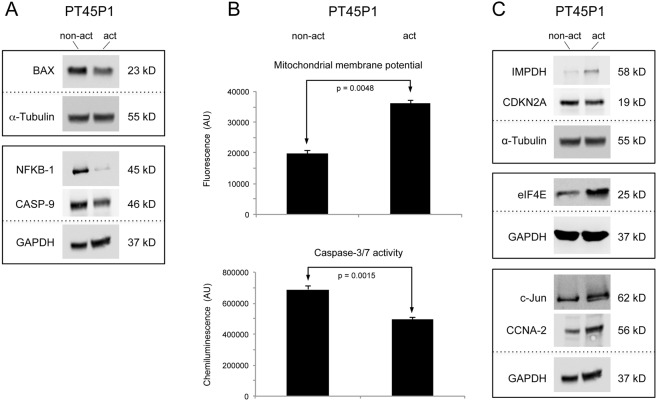
The impact of PSC secretome on PDAC cells. Serum-starved PT45P1 cells were treated with secretome from non-activated or activated PSC; the abundance of key molecules of cell apoptosis and viability were measured in the PT45P1 cellular proteome by using Western blots (**A**). Variations in apoptosis were studied by two different assays (**B**) measuring the mitochondrial membrane potential (top) or the caspase-3/7 activity (bottom). In (**C**), results from Western blots are shown that look at proteins relevant for differentiation.

To confirm the effect of the secretome released by activated PSCs on the apoptosis of pancreatic cancer cells, PT45P1 cells were grown for 48 h in medium conditioned with such secretome in comparison to growth in presence of secretome from non-activated PSCs. The cells were then harvested and both protein and total RNA were extracted, which were studied by either Western blot or real-time PCR analyses (Table 3). Conditioning the growth medium with the secretome of activated PSCs resulted in a down-regulation of pro-apoptotic factors, such as BAX, CASP-9, NFKB-1, NFKB-2, FASTK, and a concurrent up-regulation of the anti-apoptotic factor BCL-2, for example (Table 3; Fig. 3A). In addition, we confirmed that the tumour cells significantly decreased their apoptosis activity by measuring the mitochondrial membrane potential (representing extrinsic pathways) and caspase-3/7 activity (representing intrinsic pathways) in comparison to cells treated with non-activated PSC secretome (Fig. 3B). Mitochondrial membrane potential inversely correlates with apoptotic activity. Both its increase and the decrease of caspase-3/7 activity indicate a reduction of apoptotic activity. These findings confirm the functional prediction based on the protein variations observed on the antibody microarrays. Furthermore, also the functional prediction based on the protein variations detected in the PSC secretomes could be corroborated.

**Table 3:** Expression of genes grouped according to biological role.

Protein Name	Description	Fold-change	
		Microarray	qRT-PCR
**APOPTOSIS**			
***Extrinsic pathway***			
CFLAR	CASP8 and FADD-like apoptosis regulator		−1.13550
FAS	Fas cell surface death receptor	0.363	
FASTK	Fas-activated serine/threonine kinase	−0.871	
IFI27	Interferon alpha-inducible protein 27, mitochondrial		−1.81924
NFKB1	Nuclear factor of kappa light polypeptide gene enhancer in B-cells 1	−0.858	−0.76844
NFKB2	Nuclear factor of kappa light polypeptide gene enhancer in B-cells 2 (p49/p100)	0.465	
*Intrinsic pathway*			
BAX	BCL2-associated X protein	−0.578	−1.29834
BCL2	BCL2-like 2	0.295	1.84890
Caspase 9	Caspase 9, apoptosis-related cysteine peptidase	−0.321	−2.43964
**DIFFERENTIATION**			
APCS	Serum amyloid P-component	−0.481	1.04006
CD81	CD81 antigen	−0.797	
IGHM	Ig mu chain C region	−1.012	
LMNA	Prelamin-A/C	−0.497	−0.30779
POU5F1	POU domain, class 5, transcription factor 1	−0.661	
RPS19	40 S ribosomal protein S19	−0.756	−1.01396
RUNX3	Runt-related transcription factor 3	−0.486	
**ONCOGENES/TUMOR SUPPRESSORS**			
ATF3	Cyclic AMP-dependent transcription factor ATF-3		−7.76328
BRCA1	Breast cancer 1, early onset	−0.492	
CDKN1A	Cyclin-dependent kinase inhibitor 1A (p21, Cip1)	0.411	1.47768
CDKN1C	Cyclin-dependent kinase inhibitor 1C (p57, Kip2)	0.393	0.47853
CDKN2A	Cyclin-dependent kinase inhibitor 2A	−0.512	
CDKN2B	Cyclin-dependent kinase 4 inhibitor B		−2.93494
EP300	E1A binding protein p300	−0.772	
IRF7	Interferon regulatory factor 7	−0.772	
JUN	Jun proto-oncogene	0.397	0.12186
RB1	Retinoblastoma 1	−0.497	−1.03288
**SIGNALLING**			
GNAS	GNAS complex locus	0.308	
HSPA8	Heat shock 70 kDa protein 8	0.441	2.33486
IFNGR1	Interferon gamma receptor 1	0.523	
MAPK10	Mitogen-activated protein kinase 10	0.401	
MAPK3	Mitogen-activated protein kinase 3	−0.516	−1.03526
S100A4	S100 calcium binding protein A4	0.310	
S100A6	S100 calcium binding protein A6	−1.444	
S100A8	S100 calcium binding protein A8	0.382	
S100A9	S100 calcium binding protein A9	−0.733	
SOCS1	Suppressor of cytokine signalling 1	−0.932	
VEGFC	Vascular endothelial growth factor C	0.458	
**CELL CYCLE**			
BUB1	BUB1 mitotic checkpoint serine/threonine kinase	0.489	
CCNA2	Cyclin-A2	0.745	3.96320
CCNB1	Cyclin B1	0.448	
CD72	CD72 molecule	0.442	
CDKN1C	Cyclin-dependent kinase inhibitor 1C	0.393	
GAS1	Growth arrest-specific 1	0.403	
**TRANSLATION INITIATION**			
EEF1A1	Elongation factor 1-alpha 1	−0.422	−1.37554
EIF2B1	Translation initiation factor eIF-2B subunit alpha	0.490	1.10445
EIF3B	Eukaryotic translation initiation factor 3 subunit B	−0.974	
EIF3I	Eukaryotic translation initiation factor 3 subunit I	−0.657	
EIF4E	Eukaryotic translation initiation factor 4E		2.20381
IMPDH	Inosine-5′-monophosphate dehydrogenase 2		1.378724

To determine whether secretome from activated PSCs was also inhibiting tumour cell differentiation, as predicted, we examined the expression of particular differentiation proteins. The mRNA levels of LMNA and RPS-19, for example, were reduced in PT45P1-act (Table 3). Furthermore, most proteins of the purine metabolism and translation initiation were up-regulated (e.g., IMPDH, eIF2B-1 and eIF4E) (Table 3; Fig. 3C). Expression profiling further indicated up-regulation of proto-oncogenes or oncogenes, such as c-Jun, and down-regulation of tumour suppressor gene RB-1. Cell-cycle regulator genes that activate cell-cycle progression, for example CCNA-2, CCNB-1, CD-72, and GAS1, were also up-regulated, whereas expression of cell-cycle inhibitors, including CDKN2A, decreased (Table 3; Fig. 3C).

**Figure 4:**
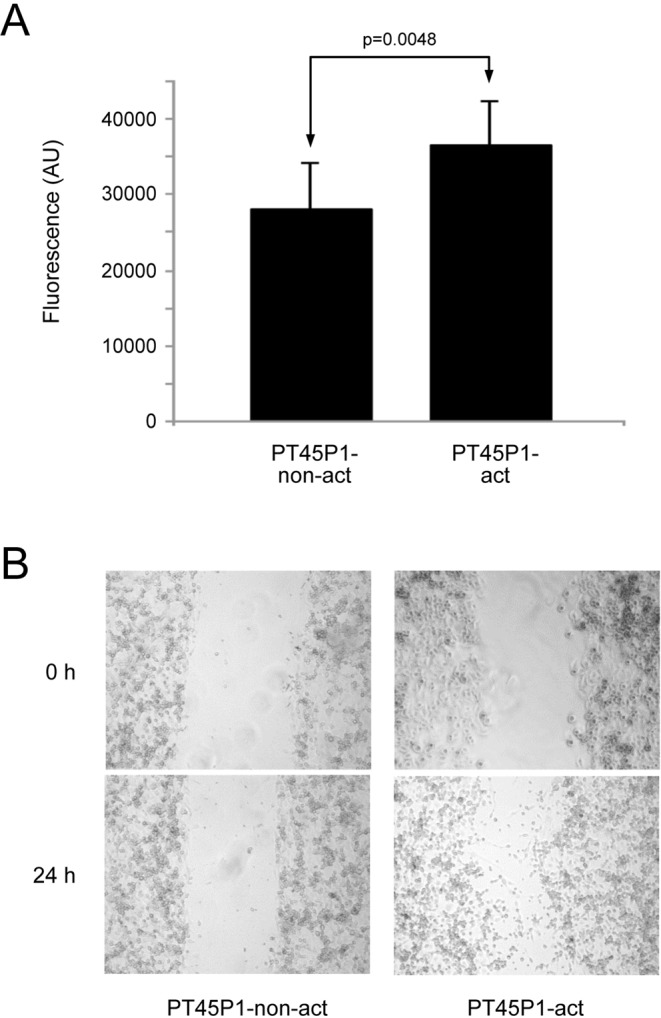
Proliferation and migration assays with PT45P1 cells upon growth in activated or non-activated PSC secretome. In (**A**), the variation of proliferation after 48 h is shown. Below (**B**), the effect on cell migration after 24 h is presented using a scratch assay.

Finally, we also studied the effect of secretome from activated PSCs on the proliferation and migration of PT45P1 cells. Measuring proliferation by an assay that is based on quantifying the cellular DNA content demonstrated that exposure to secretome of activated PSCs significantly increased the rate of proliferation of the tumour cells (Fig. 4A). For an initial analysis of migration capacity, a wound healing scratch assay was employed (Fig. 4B). Cell migration was significantly increased in PT45P1 cells after a 24 h treatment with secretome from activated PSCs as compared to secretome from non-activated PSCs (p < 0.05).

### Activated PSCs promote pancreatic tumour cell growth through the eIF4E pathway

**Figure 5:**
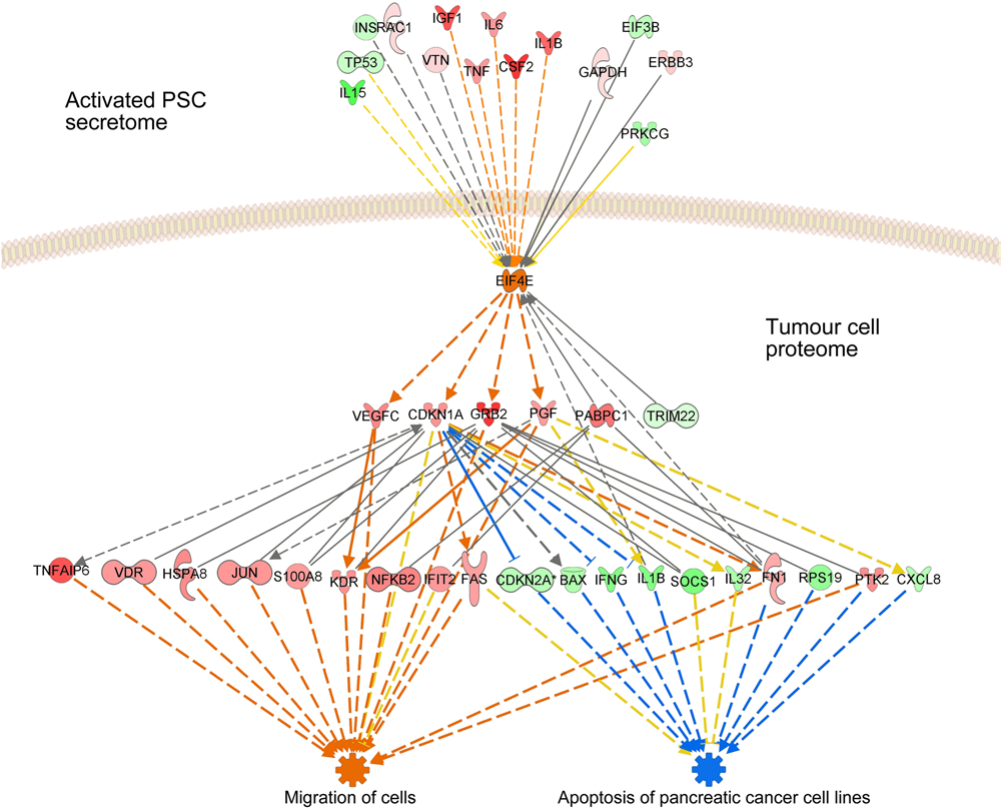
Schematic representation of the regulative function of eIF4E. Using the IPA analysis software, effects are shown of proteins of the secretome of activated PSCs on eIF4E (upstream factors) as well as the effects of eIF4E on intracellular proteins of the tumour cells. Red indicates an increase, green a decrease in abundance compared to control conditions. Orange stands for activation, blue for inhibition.

**Figure 6:**
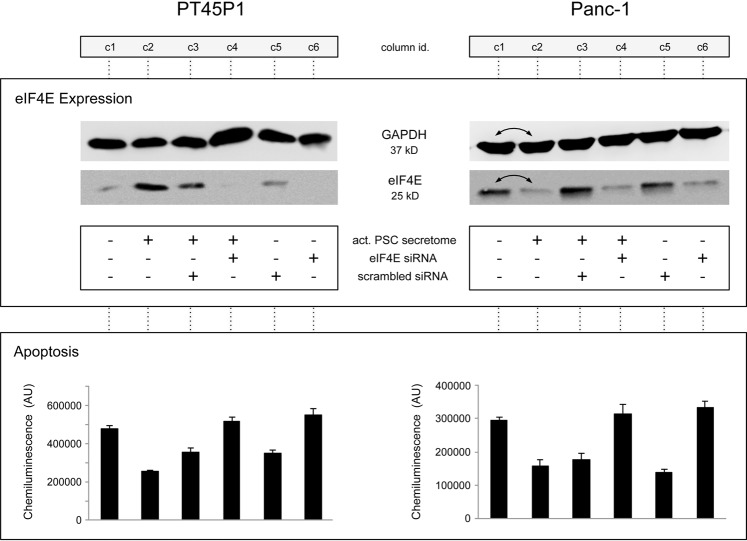
The effect of knocking down eIF4E protein expression by small interfering RNA. Tumour cells PT45P1 and Panc-1 were transfected with 100 nM of an eIF4E-specific siRNA or a control siRNA of scrambled sequence for 48 h, followed by serum starvation overnight. Subsequently, they were grown in presence or absence of activated PSC secretome for 24 h. The effect of the various conditions on eIF4E is shown, determined by Western blotting. Also, the results of caspase-3/7 assays are shown for each column (c1 to c6). Note: the two lanes c1 and c2 are exchanged in the Western blots generated from Panc-1 cells.

A pathway analysis of the proteins that exhibited variations in PT45P1-act indicated that the eukaryotic translation initiation factor 4E (eIF4E) could act as an upstream regulator of many of the proteins that showed changes in their expression. At the same time, eIF4E was found to be affected by various proteins of the secretome of activated PSCs (Fig. 5). eIF4E has been reported to affect oncogenes and growth factors promoting PDAC^30,31^ and was found strongly up-regulated in tumour cells. Therefore, we postulated that regulation of the eIF4E signalling pathway might be involved in functional changes observed for PT45P1-act. If so, the effect of conditioning the growth medium of tumour cells with the secretome of activated PSCs should be suppressed by inhibiting eIF4E expression. To test this, two tumour cell lines – PT45P1 and Panc-1 – were transfected with *EIF4E*-siRNA or a control-siRNA of scrambled sequence and cultured for 24 h. Subsequently, the cells were grown in medium for another 24 h that was supplemented with the secretome of activated PSCs or relevant control conditions (Fig. 6).

**Figure 7:**
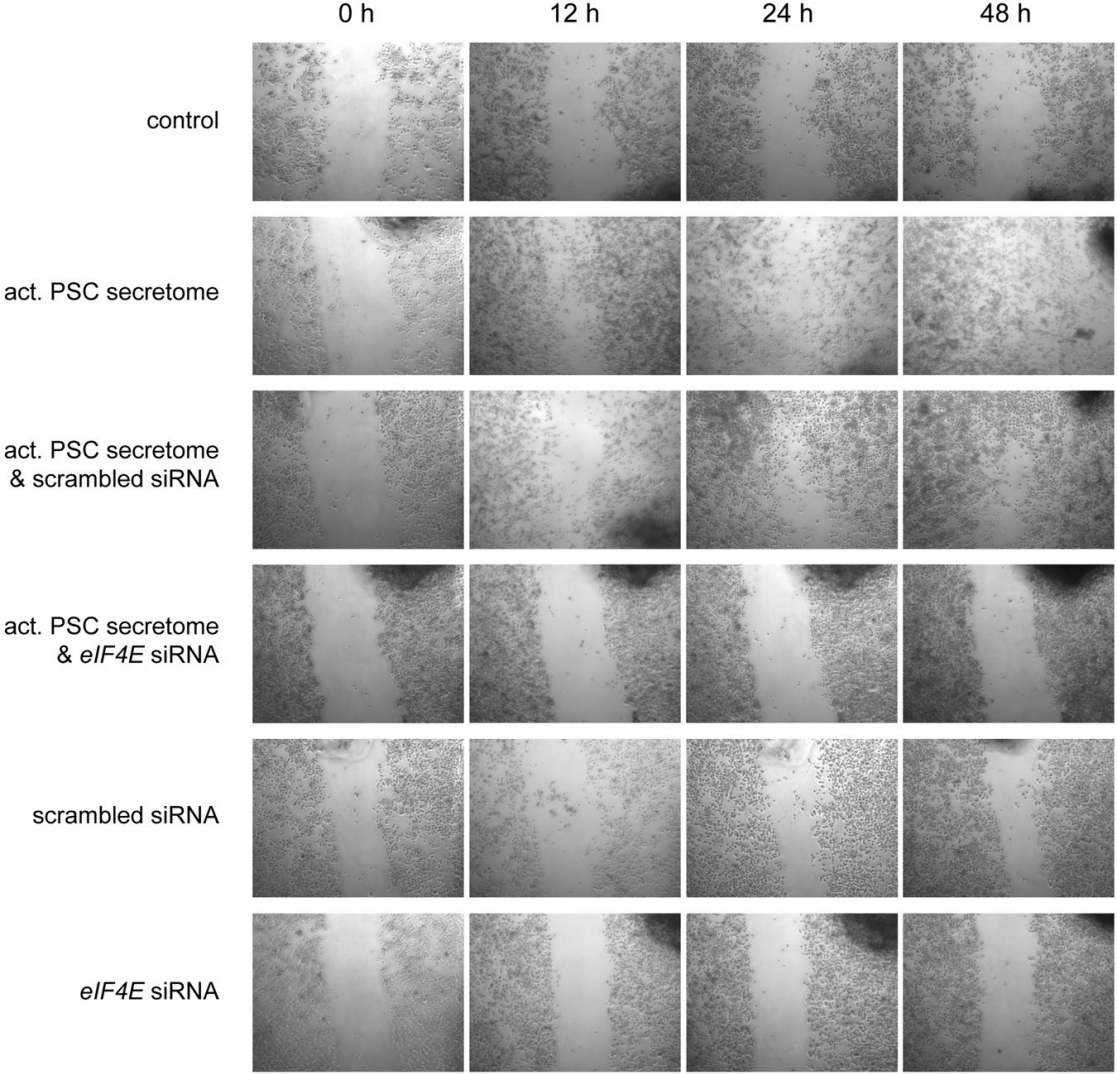
Transient silencing eIF4E in Capan-1 pancreatic cancer cells inhibits cell migration. Capan-1 cells were transfected with 100 nM of either eIF4E-specific siRNA or a control siRNA of scrambled sequence for 48 h. Cells were serum starved overnight and either left untreated or treated with activated PSC secretome. A gap was generated by physically scraping off cells. The gap was inspected at different time intervals of up to 48 h at the growth conditions indicated in the figure.

Western blot analyses showed that there was little eIF4E expression in absence of secretome, while the presence of activated PSC secretome led to a strong induction of eIF4E expression (Fig. 6, columns c1 & c2). Suppression of eiF4E expression was very specifically achieved with the *EIF4E*-siRNA, counteracting the secretome induction (Fig. 6, c4). An siRNA with unspecific sequence had no such effect (Fig. 6, c3). Functionally, the effect of inhibiting eIF4E expression was tested by looking at apoptosis, employing the caspase-3/7 assay as a means for measuring the combined effect of siRNA and conditioned medium. In accordance with the eIF4E levels, apoptosis changed as expected (Fig. 6). The same is true also for proliferation (not shown). For both apoptosis and proliferation, the cellular response to the addition of secretome from activated PSCs could be reversed by silencing specifically *EIF4E*. Next, we analysed whether inhibition of eIF4E expression also impacted tumour cell migration. For this assay, another PDAC cell line was used – Capan-1 – since it acts as a model for pancreatic cancer metastasis. In a scratch assay, cell growth in serum-free medium resulted in little migration. Addition of secretome from activated PSCs changed this strongly; within 48 h, the gap was completely filled with cells (Fig. 7). Transfecting the cells with a scrambled siRNA did not make a difference. However, upon transfection with an *EIF4E*-specific siRNA, the migration stopped entirely, although the cells were grown in the presence of secretome. Inhibition of eIF4E expression strongly suppressed the invasive capacity of the Capan-1 cells, suggesting that elevated eIF4E expression is associated with positive regulation of cell migration.

## Discussion

Proteins secreted by stroma or tumour cells represent a major part of the molecules involved in intercellular communication, cell adhesion, motility and invasion^32^. We had previously examined the effect of proinflammatory factors on the biological functions of PSCs and identified TNF-α is the prime molecule responsible for their activation^6^. In the study reported here, we followed up this work by looking at regulatory mechanisms in tumour cells that are governed by molecules released from activated PSCs. Comparing the secretome of activated and non-activated PSCs identified proteins that were secreted by the former cells but not their quiescent equivalent. These proteins are predominantly involved in biological functions that are associated with cancer pathologies. We confirmed the functional predictions by growing tumour cells in media supplemented with PSC secretome. Simultaneously, we looked at the variations occurring in the proteome of such tumour cells. The eukaryotic translation initiation factor 4E (eIF4E) was found to act as a connecting regulative factor. We used *EIF4E*-silencing experiments to corroborate its involvement in the actual functional regulation.

A number of signalling pathways lead to PSC activation in response to cytokines. During inflammation, cytokines and growth factors like TGF-ß1, PDGF, interleukin-1ß (IL-1ß), IL-6 and TNF-α are released in the pancreatic tumour microenvironment causing stimulation and activation of PSCs^33^. Evidence from this laboratory and others suggests that TNF-α is a key element in PSC activation^6,34–36^. In fact, TNF-α is known to be highly upregulated in pancreatitis with severe pancreatic fibrosis^37^. TNF-α is also a well-known mediator in the transformation and differentiation of myofibroblasts^38^. In pancreatic tumours the transformation is usually associated with induced proliferation of PSCs, expression of α-SMA, and synthesis of fibronectin. TNF-α is an inflammatory cytokine produced mainly by macrophages/monocytes as well as tumour cells during acute inflammation and responsible for a diverse range of signalling events within cells^39^. For this reason, we focussed our analysis on activation mediated by TNF- α.

Among the proteins detected in the secretome of activated PSCs were multiple cytokines and growth factors. Molecules such as IL-1ß^40^, S100 calcium binding protein A4 (S100-A4)^41^, TNF-α^42^, connective tissue growth factor (CTGF)^43^ and activin^44^ have been implicated to be involved in proliferation, migration or invasion of pancreatic cancer cells. IL-1ß, for instance, is known to up-regulate the expression of vascular cell adhesion protein 1 (VCAM-1) which mediates pro-metastatic tumour-stromal interactions^45^, promoting cancer cell adhesion and metastases^46^. PSCs also released platelet derived growth factor (PDGF)^11^, vascular endothelial growth factor (VEGF)^12^, TGF-β^47^ and cyclooxygenase-2 (COX-2)^48^, which promote cancer cell growth. Other molecules secreted by activated stellate cells were fibroblast growth factor 1 (FGF1), IL-6, IL-4 and insulin-like growth factor I (IGF-1). Proliferation of pancreatic cancer cells is known to be mediated by PDGF and FGF1 signalling pathways^11^. Studies have shown that these pathways are influenced by the interplay of tumour cells and their microenvironment^49^. Furthermore, IL-1ß, IL-6 and TNF-α are present in the microenvironment of cancer, contributing to metastasis and drug resistance^36^. The increased IL-6 secretion by activated stellate cells strongly suggests a role in regulating cell growth in the cancer microenvironment. Another protein identified was plasminogen activator inhibitor 1 RNA-binding protein (SERPINE), whose deregulation has been associated with the progression of various cancers, including PDAC^50^. In addition, SERPINE co-localizes with hypoxic regions in tumours^51^. Also thrombospondin 3 (THBS-3) was specific for activated PSC secretome. THBS-3 is a member of adhesive glycoproteins that mediate cell-to-cell and cell-to-matrix interactions. THBS-3 binds to ECM proteins, including fibronectin, laminin and type V collagen^52^. Other ECM proteins that were found, such as FN1 and Col1, have previously been reported to be involved in pancreatic cancer progression. Their high abundance in the secretome of activated PSCs suggests a *de novo* induction of expression and subsequent secretion upon PSC activation. The release of ECM proteins represents a pivotal role of activated PSCs in tumorigenicity. They have important roles in resistance to anticancer drugs and cell proliferation of pancreatic cancer cells^53^.

Next to studying the content of the PSC secretome and predicting functional consequences it may have on recipient tumour cells, we performed incubations of PDAC cells in growth medium conditioned with PSC secretome from activated and non-activated cells. Intracellular expression was analysed by means of the antibody microarray and qRT-PCR. As a response to growth in presence of activated PSC secretome, we found a significantly inhibition of apoptosis and a simultaneous promotion of tumour cell migration. Furthermore, proliferation of the pancreatic cancer cells was induced. This fits very well to the predictions made on the basis of the variations in the PSC secretome. This result suggests a direct impact on the tumour cells by activated PSCs in addition to their involvement in the desmoplastic events within the tumour microenvironment. In response to activated PSC secretome, apoptotic factors of the extrinsic and intrinsic pathway were regulated. Such an effect was not observed with non-activated PSC secretome. Among proteins that exhibited significant differences in abundance was nuclear factor kappa B subunit 1 (NFKB-1). This protein is not expressed by normal cells, but strongly expressed in a number of human cancers including non-small cell lung carcinoma, pancreatic cancer, colon cancer, prostate cancer, breast cancer, bone cancer and brain cancer^54^. In pancreatic cancer cells, reduced expression of NFKB-1 activating molecules can obstruct tumour progression *in vitro* and *in vivo*^55^. In addition, NFKB-1 prompts the expression of inflammatory cytokines, adhesion molecules, key enzymes in the prostaglandin synthase pathway (COX-2), nitric oxide (NO) synthase and angiogenic factors. Furthermore, by inducing anti-apoptotic genes (e.g. BCL-2), it promotes survival in tumour cells and epithelial cells targeted by carcinogens^56,57^.

Many proteins involved in tumour cell differentiation, such as ribosomal protein 19 (RPS-19), lamin A/C (LMNA) and immunoglobulin heavy constant mu (IGHM), exhibited decreased expression upon cell treatment with activated PSC secretome. The results support previous observations that PSCs can reduce differentiation of PDAC cells^58–60^. The molecular changes triggered in the tumour cells could be responsible for dissuading cells from both differentiation and apoptotic processes^61,62^. In response to treatment with secretome from activated PSCs, expression profiling further indicated down-regulation of proto-oncogene or oncogenes, such as the retinoblastoma-associated tumour suppressor gene *RB-1*. Cell-cycle regulators that activate cell cycle progression, such as cyclin-A2 (CCNA-2), were also up-regulated, whereas expression of cell-cycle inhibitors, including the CDK-inhibitor 2 A (CDKN2A), decreased in PDAC as compared to controls. RB-1 is a crucial regulator of appropriate cell cycle progression, including G1 to S and G2 to M phase transitions^63^. The activity of RB-1 is mainly regulated by the upstream CDKN2A/CCND-1 pathway^64^. In accordance with the important role of RB-1 as a cell cycle regulator, RB-1 deregulation is observed in multiple types of cancers^65^. Functional loss of RB-1 causes accelerated, E2F-transcription factor 1 (E2F1) mediated transactivation, followed by uncontrolled cell cycle progression^66^.

Even though the role of PSCs in the microenvironment is well-established^10,11^, the cellular mechanisms mediating crosstalk between PSCs and PDAC cells and the signalling molecules involved remain a complex network awaiting to be untangled. Tumour cell growth in secretome of activated PSCs caused a significant increase in the expression of proteins of translation initiation, such as translation initiation factor eIF-2B subunit alpha (eIF2B-1) and particularly eukaryotic translation initiation factor 4E (eIF4E). Protein eIF4E triggers the synthesis of a variety of molecules involved in cell growth, proliferation and invasion, including the cell cycle regulatory protein cyclin D1 (CCND-1)^67^, the transcription factor c-Myc, growth factors such as the vascular endothelial growth factor (VEGF) and fibroblast growth factor 2 (FGF-2)^68^, as well as the anti-apoptotic protein MCL-1^69^. Recent studies have demonstrated that progression and resistance to standard therapies in tumour is closely related to the eIF4E pathway^30,31,70^. In our analysis, we confirmed that eIF4E acts as a key signalling molecule that mediates crosstalk between PSCs and tumour cells. In a positive feedback cycle, eIF4E expression further promoted its own expression. Additionally, eIF4E increased tumour cell motility and inhibited pancreatic cancer cells apoptosis. All these effects were effectively blocked by *EIF4E*-siRNA, confirming the essential role of eIF4E in PSC-tumour communication. These results are intriguing given that constitutive activation of eIF4E is commonly observed in tumours and the molecule is a validated target for therapy of solid cancers, including pancreatic cancer^30,31^.

In conclusion, we identified proteins secreted by PSCs, which had been activated by TNF-α, that regulate the growth and proliferation of cells in damaged pancreas via paracrine and autocrine mechanisms. The molecular communication by means of the secretome promoted pancreatic cancer cell proliferation, migration and inhibited apoptosis through eIF4E activation. Our findings provide novel evidence for the modulation of pancreatic cancer cells by PSCs in an inflammatory environment. Although further efforts are required to elucidate in completion the biological functions triggered by the secretome factors, one pathway of how PDAC progression and inhibition of apoptosis is achieved has derived from this analysis.

## Supplementary information

SupplFig1-2

Supplementary Dataset 1-7
