# Supplementary material for: Impact of the secretome of activated pancreatic stellate cells on growth and differentiation of pancreatic tumour cells
Source: Sci Rep. 2019 Mar 28;9:5303. doi: 10.1038/s41598-019-41740-x (PMC6438963; doi:10.1038/s41598-019-41740-x)
Supplement: Supplementary file 1 — SupplFig1-2 [file 41598_2019_41740_MOESM1_ESM.docx]

**Impact of the secretome of activated pancreatic stellate cells**

**on growth and differentiation of pancreatic tumour cells**

Aseel J. Marzoq^1^, Shakhawan Mustafa^1,2^, Luzia Heidrich^1^, Jörg D. Hoheisel^1^ and Mohamed Saiel Saeed Alhamdani^1^*

^1^ Division of Functional Genome Analysis, Deutsches Krebsforschungszentrum (DKFZ),

Im Neuenheimer Feld 580, D-69120 Heidelberg, Germany

^2^ Kurdistan Institution for Strategic Studies and Scientific Research, Kurdistan Region, Iraq

****Corresponding author***

Dr. rer. nat. Mohamed Saiel Saeed Alhamdani
Functional Genome Analysis/B070
German Cancer Research Center (DKFZ)

Im Neuenheimer Feld 580
69120 Heidelberg, Germany
phone: +49 6221 42-2718
fax:      +49 6221 42-4687
[m.alhamdani@dkfz.de](mailto:m.alhamdani@dkfz.de)
[www.dkfz.de](http://www.dkfz.de/)


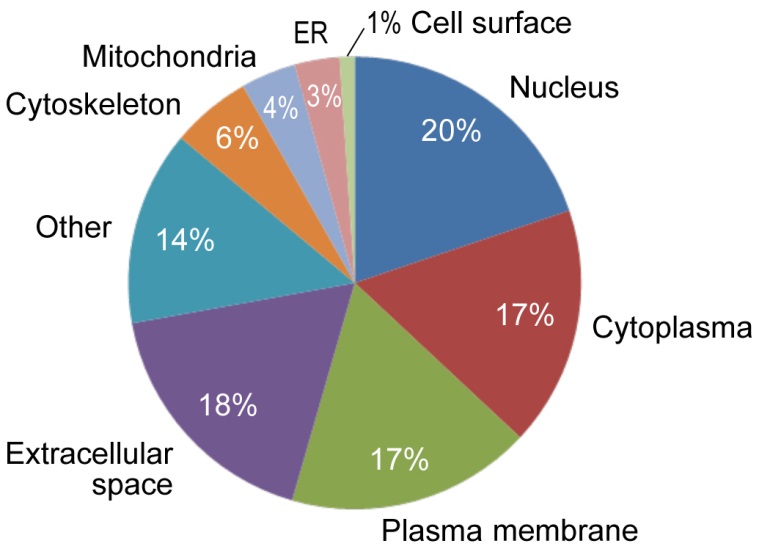


**Supplementary Figure 1.** Annotation of the proteins secreted specifically by activated PSCs.

**
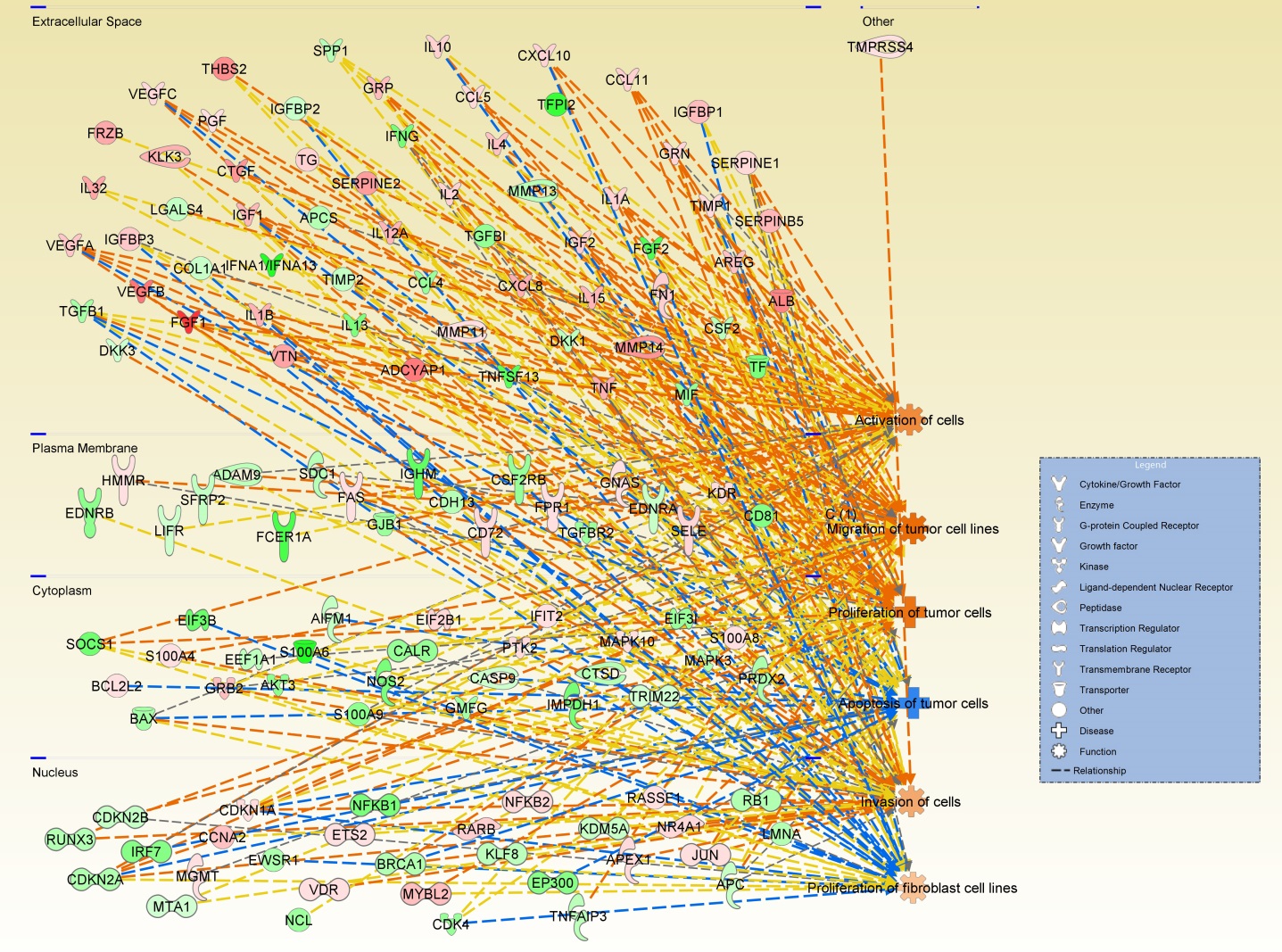
**

**Supplementary Figure 2.** Ingenuity functional prediction based on the protein variations observed in activated PSC secretome. The proteins are expected to either activate (orange) or inhibit (blue) functions like invasion, proliferation, apoptosis, migration and activation of cells. The corresponding proteins, on which the prediction was based, are also depicted. They are labelled red for higher and green for lower abundance in activated PSC secretome compared the abundance in non-activated PSC secretome. The data were analysed using Ingenuity Pathway analysis tool IPA (QIAGEN Inc., https://www.qiagenbioinformatics.com/products/ingenuity-pathway-analysis).
